# Key stakeholders’ views, experiences and expectations of patient and public involvement in healthcare professions’ education: a qualitative study

**DOI:** 10.1186/s12909-022-03373-z

**Published:** 2022-04-22

**Authors:** Megan Cullen, Cathal Cadogan, Susmi George, Siobhan Murphy, Siobhan Freeney, Robbie Fitzpatrick, Judith Strawbridge

**Affiliations:** 1 School of Pharmacy and Biomolecular Sciences, Royal College of Surgeons in Ireland, 123 St Stephen’s Green, Dublin, Ireland; 2 Beaumont Hospital Pharmacy Department, Beaumont Hospital, Beaumont, Dublin 9, Ireland; 3 School of Pharmacy and Pharmaceutical Sciences, Trinity College Dublin, Dublin, Ireland; 4 Department of Surgery, RCSI Education and Research Centre, Royal College of Surgeons in Ireland, Beaumont Hospital, Beaumont, Dublin 9, Ireland; 5 Patient and Public Representative, Dublin, Ireland

**Keywords:** Health professions education, Patient and public involvement, Patient Educator, Patient Partner

## Abstract

**Background:**

Patients and the public have an integral role in educating healthcare professionals. Authentic partnerships between higher education institutions and patients and the public are essential. This study examined key stakeholders’ views, experiences and expectations of patient and public involvement (PPI) including the nature of the involvement and requirements for partnership.

**Methods:**

Purposive and snowball sampling was used to recruit key stakeholders, including patients and members of the public involved in health professions education, and academics interested in PPI. Focus groups were held with patient and public participants, providing the opportunity to gain multiple perspectives in an interactive group setting. Academics with an interest in PPI were interviewed using a semi-structured approach. Topic guides were derived from the literature and piloted prior to data collection. Focus groups and interviews were conducted until data saturation was achieved. All data was audio-recorded, transcribed, anonymised and thematically analysed.

**Results:**

Four focus groups were conducted involving 23 patient and public participants (median number of participants per focus group of 6). Nine interviews were conducted with academics (face-to-face [*n* = 8] or by telephone [*n* = 1]). Five themes were developed: previous experiences of PPI, training requirements, challenges/barriers to PPI, facilitators of PPI and future ideas for PPI. All participants held positive views of the value of PPI. Participants had mixed views in terms of training, which depended on the level of involvement, but similar views on the challenges and facilitators for PPI in education. There was agreement that PPI requires institutional vision and investment to build strong relationships and a culture of PPI best practice.

**Conclusions:**

There is a need for more strategic and formal involvement of patients and the public to ensure that that PPI becomes sustainably embedded in health professions education.

**Supplementary information:**

The online version contains supplementary material available at 10.1186/s12909-022-03373-z.

## Background

Patients and the public have a long-standing input in the education of healthcare professionals. Initially the nature of this engagement was relatively passive [1]. Increasingly, patients and the public are more actively involved in education, through storytelling, assessment and curricula design [2]. Towle, et al. explored different levels of PPI in education ranging from paper-based activities, to co-teaching, co-designers of the curricula and sustained involvement in PPI at an institutional level [3]. They built on Tew, at al.’s ‘Ladder of Involvement’ to produce a taxonomy which measures the depth and impact of PPI in education [3, 4]. It has been demonstrated that such involvement has the capacity for motivating students, demonstrating the relevance of learning and encouraging the development of key professional skills (e.g. communication, fostering empathy) [5, 6]. Patients and the public have an integral role in educating healthcare professionals, and their involvement is considered essential for high quality education [7].

The first structured PPI programme was introduced in medicine in the 1960s with the development of ‘simulated patients’ [8]. These programmes successfully addressed problems encountered in teaching clinical skills and demonstrated student acceptability and short-term effectiveness [1, 3, 8]. PPI was later introduced to healthcare professions outside of medicine, such as pharmacy [9, 10], physiotherapy [11], occupational therapy [12] and dentistry [13], with studies reporting higher levels of involvement of patients in education [11].

More recently, there has been a noted increase in the diversity of roles and an extension of PPI to postgraduate and continuing professional education. A systematic review of patient involvement in medical education reported that patients are increasingly being more involved in student selection, summative assessment and curriculum development [2]. Similarly, an observational study reported PPI in student recruitment, student assessment, programme management, course evaluation, curricula design across multiple universities and training establishments [4, 7]. A review reported a lack of resources and a need to move from isolated initiatives to sustained and authentic partnership with patients at an institutional level [3]. A systematic review to identify contextual factors and strategies to enable optimal PPI in the design, delivery and evaluation of health services reported that half of the included studies demonstrated a low level of PPI [7]. There is also a wide variation in the manner and extent of PPI in the education of healthcare professionals [6].

Recommendations have been made to identify more ways of involving patients in education [14]. However, consensus on how to best optimise PPI is currently not available [15]. This lack of consensus was echoed in a recent Best Evidence Medical Education (BEME) collaboration systematic review which concluded that, while the benefit of PPI is well established in the literature, there is a lack of an underpinning conceptual basis to translate theory into practice [6]. While recent studies have shown that patient involvement can effectively deliver essential practical skills to students and enrich healthcare education, the extent to which patients are involved at an institutional level has not improved, nor have the outcomes of PPI interventions progressed [6]. One systematic review reported that longitudinal institutional incorporation, resource support, patient recruitment and training and clear faculty commitment are required to support sustainable PPI in education, however more information is required to update PPI frameworks and to identify patient needs and roles in education [2]. Past studies have also shown gaps in the literature in relation to ethical issues, long- and short- term outcomes, psychological impact and key procedural contributors such as recruitment, selection and preparation [6, 16, 17]. The need of participant training is an important concept to address in PPI. While training can be beneficial some studies have suggested that training could prevent PPI participants from being the general representative of that population [18].

A greater knowledge of key stakeholder views, requirements and expectations are essential to the incorporation of sustainable PPI at an institutional level [4, 19]. The aim of this study is to obtain key stakeholders views, experiences and expectations of PPI in healthcare education that will form recommendations for the development of deep and sustainable PPI in health professions education.

The objectives of this study are to:

- Explore how key stakeholders think patients and the public need to be involved in educating healthcare professionals;
- Examine what motivates people to become involved in PPI and what extent are they willing to be involved;
- Explore what key stakeholders think is required for deep and sustained patient and public involvement in healthcare education.

## Research methodology

### Study design

A qualitative study was conducted comprising focus groups and semi-structured interviews. This study received ethical approval from the Royal College of Surgeons in Ireland (RCSI) Research Ethics Committee (REC1609). Focus groups were selected for patients and the public to gain multiple perspectives in an interactive group setting. Focus groups were chosen to encourage participants to explore both individual and shared experiences and views [20]. An interview approach was selected for members of faculty who have experience with PPI to identify views and attitudes to PPI in education. The Consolidated criteria for Reporting Qualitative research (COREQ) qualitative checklist was used to aid reporting of study methods, context of the study findings, analysis and interpretation (Additional file 1) [20].

### Sample selection and recruitment

Purposive and snowball sampling was used to identify participants [21]. Two cohorts of participants were involved in this study: patient and public participants (cohort 1) and members of university faculty (cohort 2). Patient and public participants were included in this study to involve people with experiences of health services, patient advocates, carers, and family members. Simulated patients and actors were also defined as public participants.

Patients were invited by a gatekeeper to participate from a list of patients who are involved in education in the university. Patient and public participants were also invited via patient advocacy groups such as Aware Ireland (provides support for people experiencing depression or bipolar disorder), Parkinson’s Ireland and the Multiple Sclerosis Society of Ireland. Advocacy groups were contacted and study details were circulated to members, asking for those interested in participating to email a member of the research team. Staff were invited from within the university. A selected number of academics were invited from outside the university based on having been identified as leading on PPI through literature review, with one participant located outside of Ireland, in the United Kingdom.

### Data collection

Participants were invited to attend focus group discussions or interviews. Topic guides were developed based on a literature review and piloted prior to data collection by MC with two individuals external to the research team to ensure clarity of questions and that relevant data were collected (Additional file 2) [3, 7, 15]. Two topic guides were designed, one for focus groups with patient and public participants and another for interviews with members of faculty. The focus group topic guide covered past experiences in PPI, motivation for being involved in PPI, how could academic institutions best recognize their contributions, views on training for PPI and facilitators and barriers to their involvement in education. The interview topic guide explored how patients and the public can best be involved in education, training requirements, motivation to be involved (for both patients and the public and staff) and facilitators and barriers for academics becoming involved or more involved in PPI. Focus groups and interviews were audio-recorded and transcribed by SG. Field notes were also taken during focus groups and included in the analysis. Participants could then review, edit or erase their transcript up to 14 days after they were transcribed, after which personal identifiers were removed from transcripts. Data were collected until data saturation was achieved. This was defined as the point at which no new themes or codes emerge from the data [22].

### Data analysis

Thematic analysis was employed with an inductive approach and worked within a constructivist epistemology, in accordance with published guidance [23–25]. A six step process was employed, as developed by Braun and Clarke [24]. This method involved familiarization with the data, generating initial codes, searching for themes, reviewing themes, defining and naming themes and documenting the results. Reflexivity was also considered, where researcher biases and assumptions were explored prior to data collection and during data analysis. Reflexive conversations were undertaken during research meetings on author views and acknowledgements were made on respective author backgrounds and how this may impact analysis. Author biases was recognized and recommended approaches were utilized, such as double-coding and iterative interpretation, to aid rigorous and transparent analysis [26]. Two investigators (MC, SG) conducted primary coding, codes were then reviewed with other members of the research team (JS, CC) and resolved discrepancies by consensus discussion. SF and RF were study patient collaborators and were involved in data interpretation. The researchers discussed various themes and verified relationships between themes and data codes. Some of the quotes fell under multiple themes as they demonstrated relevance to more than one area. A consensus of all collaborators was used to develop the final structure of relationships between themes and select representative data to illustrate the themes.

Data from the interviews and focus groups were triangulated using a convergence coding matrix. This involved reviewing the findings from each cohort to determine whether there was agreement, partial agreement, silence or dissonance between the cohorts [27].

## Results

### Participant characteristics

Four focus groups were conducted involving 23 patient and public participants (12 female). The median number of participants per focus group was six. Nine interviews were conducted with academics (five female), either face-to-face (*n* = 8) or by telephone (*n* = 1). Focus group discussions each lasted approximately 60 min and each interview lasted approximately 20 min.

Most focus group and interview participants had some degree of PPI involvement, with most of the involvement being at a low level, for example as patients or simulated patients for Objective Structured Clinical Examinations (OSCE). There was some experience with PPI with higher levels of involvement, such as workshops where patients had an opportunity to discuss their experiences with their disease and the healthcare system and give feedback to students.

### Thematic analysis code generation

Five themes were developed (Table 1). These themes comprised; previous experiences of PPI events, training, challenges/ barrier of PPI, facilitators/ enablers of PPI and future ideas for PPI activities.

**Table 1:** Themes and subthemes identified in thematic analysis

Themes	Subtheme
1. Previous experiences of PPI	Experiences and impact
2. Training	Desire for training
	Description of training
	Effect on meaningful engagement
3. Challenges/Barriers	Patient and public challenges/barriers
	Academic facilitators challenges/barriers
	Institutional challenges/barriers
4. Facilitators	Patient and public facilitators
	Academic facilitators
	Institutional facilitators
5. Future ideas for PPI	Method of Involvement (description)
	Timing/duration of activity
	Recruitment methodology

### Convergence coding matric for contextual factors

A Convergence Coding Matric for Contextual Factors (Table 2) was developed to illustrate where agreement, partial agreement, silence and disagreement was observed between and within groups of participants.

**Table 2:** Convergence coding matric for contextual factors

Themes	Subtheme	Convergence Code							
		**Interview**				**Focus Groups**			
		**Agreement**	**Partial agreement**	**Silence**	**Disagreement**	**Agreement**	**Partial agreement**	**Silence**	**Disagreement**
**1. PPI experiences**	Past experiences/ impact of PPI	●				●			
**2. Training**	Desire for training				●				●
	Description of training		●				●		
	Effect on meaningful engagement		●				●		
**3. Challenges/ Barriers**	Patient and public challenges/barriers	●				●			
	Academic facilitators challenges/barriers	●						●	
	Institutional challenges/barriers	●						●	
**4. Facilitators**	Patient and public facilitators	●				●			
	Academic facilitators	●						●	
	Institutional facilitators	●						●	
**5. Future ideas for PPI**	Timing/ duration of activity	●				●			
	Method of Involvement (description)		●			●			
	Recruitment/ recruitment methodology	●				●			
**Total**		9	3	0	1	6	2	4	1

### Completeness comparison

#### Previous experiences and impact of PPI

Most participants had positive views of their previous experiences in PPI in education. Patient and public participants discussed the positive social side of the events as well as seeing the benefits for the students, whereas staff mainly focused on the positives for the students. Patient and public participants reported that their past experiences were very enjoyable. All focus group and interview participants agreed that they saw immense value of PPI events for students.

*“It’s much better learning from those who suffer from a disease than learning from a book” (focus group participant D).*

There was also a uniform expression of how much students enjoyed and engaged in such events and how it demonstrates relevance of learning for students and has a humbling effect on both students and academic staff.

*“That person really did educate all of us and remind us in terms of why we’re here and stopped us meandering really and brought us back to focus” (interview B).*

Participants also believed that it would have a beneficial on professional development and helps create equal relationships between patients, students and healthcare professionals.

*“I think that’s a wonderful educational opportunity because we are equal” (focus group participant K).*

Although overall the views about PPI were very positive, there were some concerns about getting the right participant for PPI in education.

*“There might be problems with getting the right sort of members of the public who could be involved. If we’re not very careful, we could end up with patients with very narrow and negative agendas. And we need to avoid that” (interview D).*

The issues expressed by patient and public participants concerned how they felt during the experiences. They described occasions where they felt uncomfortable or embarrassed.

*“What I found disconcerting at that stage, was not the student at all, but he was being asked a lot of questions, by one of the consultants … poor young fellow kept getting it all wrong because, you could see he was panicking and it was just embarrassing for me” (focus group participant V).*

Other participants expressed frustration at their lack of involvement in the events.

*“You need to have some acknowledgement of you as a person…. I would appreciate that I would be asked by the examiners, ‘well how was that for you?’ or ‘have you anything to say?’” (focus group participant K).*

### Training for PPI

#### Desire for training

There was a dissonance in views for training of patient and public participants within both focus groups and interviews. Most focus group participants described that while they would not mind undergoing formal training, they did not believe it was necessary for activities where they are to share their experiences with the disease. However, some patient and public participants reported that they would need training to participate in education. Similarly, while all staff believed that some training of participants was required, there was a dissonance in views to what degree of training a participant needs to receive. The majority believed that training is needed but more so for role clarification, guidance and to establish participants expectations of events.

*“I think just a brief overview is important. Then depending on what teaching they are involved in, that will determine what additional training. I suppose the way I’d see it is you have your baseline that everybody gets across the board, and that’s your orientation, professionalism and just do no harm. Then you add on to those whatever extra bits are needed for that specific role, be it teaching the simulated patient or patients how to give feedback to the students appropriately” (Interview A)*.

#### Description of training

The consensus was that there needs to be guidance rather than for formal training for most PPI in education. Ideally, this would be provided in an informal environment in small groups or one-on-one meetings with the relevant staff member prior to the activity. Role-play and workshop style sessions were also considered beneficial, with some suggesting that they could observe PPI to learn through experience or participate in a “buddy” system. These may provide opportunities to empower people to become more involved.

*“You say PPI events are already here, it’s happening, there are patients involved. If we could see them, I know what would help me is if I could see… come along to someone else’s PPI session” (focus group participant S).*

#### Effects on meaningful engagement

There were concerns that training might result in patient and public participants no longer being representative.

*“I think it’s a balance. You don’t want to lose that rawness of being the patient and that real life experience, but also you don’t want to be going off in tangents, or all around the place or being irrelevant to what the student really needs to get out of it. So, I think there does need to be some structure around, some element of training or some kind of a toolkit to help” (focus group participant V).*

Most academic views echoed this statement and expressed concern if training is provided beyond general information on the organization and running of the event.

*“If a patient is coming in to tell their story about their experience, then probably you need less training for that because you don’t want to shape the patient’s story either. You want it to be theirs and for them to take ownership of it” (interview F).*

Staff and patients also suggested that training on PPI needs to be provided for academics to teaching them about building relationships to optimize PPI in education.

*“It is not just about having the right people, patients. It’s also about training the academics and the researchers to understand what meaningful engagement is” (interview C)*.

### Challenges / Barriers for PPI

#### Patient and public challenges/barriers

Patient and public participants mainly focused on their own barriers and limitations to involvement. While many were confident in their ability to participate in education, others expressed some concerns, including challenges with public speaking.

*“I wouldn’t know much other than the experiences I was involved with, I don’t know whether that sort of thing would be enough” (focus Group participant G).*

Despite the desire to increase the current level of involvement some focus group participants believed that they would feel unequipped unless guided if involved at higher levels such as curricula design.

Availability and illness were other barriers mentioned by participants.

*“My eye condition, I’ve been struggling, since last September I’ve spent more times in A&E, more times at home in pain and even when I’m finished here guess where I’m going” (focus group participant O).*

Feeling of not meaningfully contributing was another barrier expressed by focus groups. Lack of confidence with public speaking, along with availability and illness, were also considered barriers to PPI by staff.

Multiple participants expressed concern of having the optimal PPI participants, in terms of diversity and the best fit, where certain subsets of PPI participants are difficult to recruit, for example younger adults as they may be working or have other commitments or different socioeconomic classes of participants. Patient selection was also considered a challenge for PPI.

*“You’ll always encounter a challenging person or challenging patient. That certainly needs to be managed because students are vulnerable. They’re learning. They’re being exposed as they’re developing their skills” (interview A).*

Academics also expressed concerns for patient and public participants themselves.

*“You also have to be careful and mind that person because what you don’t want is that person telling their story, reliving their trauma. They need to have been at a particular stage of their story that they can tell their story without reliving and upsetting themselves” (interview B).*

Another challenge is that PPI may give too specific of a view into a particular disease for a particular person.

*“Everyone’s illness experience is so different. So, I guess there’s a danger there of possibly that becomes too specific or that students might identify, okay, well, this is what it’s like to be a carer for person with dementia, and not realize that that’s so different for everyone or somebody might have a completely different take on that situation” (interview F).*

#### Academic facilitators challenges/barriers

Barriers for academic involvement were also discussed. Interviewees discussed the risk that PPI could be viewed as an obligation, with a lack of appreciation of the value to education.

#### Institutional challenges/barriers

Staff also discussed a number of institutional barriers to PPI. Lack of adequate resources, time, standardised frameworks and strategy for inclusion were mentioned.

### Facilitators of PPI

#### Patient and public facilitators

All participants discussed facilitators for PPI in education, however, staff also discussed facilitators for PPI at an institutional level, which was mainly silent in the focus groups. All study participants believed that payment was important to show appreciation for patient and public participation and that people will not lose money for participating or be excluded because they could not afford to participate.

*“I do think payment is important. There are people who would get involved in a voluntary basis. From a coordinators point of view, it’s much easier to get commitment when there’s a payment. I also think it values their time.” (interview A).*

Hospitality was also considered very important in terms of organizing and paying for transport to and from events and providing refreshments.

*“It’s very nice the one-4-all vouchers, the teas and coffee and all that…. you just feel you’re being well treated (focus group participant N).*

The primary intangible motivation factor for patient participants was a feeling of that they were giving back and helping to improve and advance the healthcare system.

*“Giving back and making it better for other patients, making the system better you know …more patient centric” (focus group participant V).*

The need to feel that participants are contributing meaningfully and that they themselves are also gaining from the experience was expressed as essential to achieve sustainability of events. One focus group participant expressed that their primary reason for not getting more involved was:

*“if I felt I wasn’t contributing or I wasn’t getting something out of it. Those are the main drivers” (focus group participant S).*

Most participants also said that it a great social event and something to look forward too. The PPI participants enjoy speaking and working with the students and sharing their past experiences. Most study participants had positive views towards a PPI society as a focal point of support and to add to the social aspect of PPI in education.

*“So, anything to create a community, I think would be really useful” (interview C)*.

*“You link up with a few people and you get to chat to the same people.” (focus group participant V).*

The use of formalized titles for patient and public participants was met with a dissonance in views from focus group participants and interviewees. Some patient and public participants appreciated being referred to as an educator.

*“‘Expert by experience’ Yeah I think it’s very nice. I am not one to make a big deal of it, but it is nice” (focus group participant L).*

Consistent communication with staff is important so that participants know who they are to ask for and they recognize the face when participating in PPI. Collegiality and the establishment of strong relationships among academics and PPI participants was considered necessary for sustained PPI. Having a dedicated area for PPI was also useful facility so participants know where to go.

*“I also think having a dedicated space. So again, from my experience with the simulated patients often teaching can happen in multiple different places around the college. Now we have the patient lounge in the new building. So, they always know to come to the patient lounge, and if they arrive early or whenever they arrive, the patients in the lounge can chat to each other” (interview A).*

#### Academic and institutional facilitators

Staff considered implementation of a strategic framework for organizing and hosting PPI events a major institutional facilitator of PPI. Through this framework payment of participant in accordance with their level of involvement needs to be standardised throughout all departments along with standard operating procedures (SOPs). It was also suggested that different frameworks must be developed depending on the level of involvement.

*“Different frameworks for very minimal involvement, limited engagement, more engagement and then true collaboration … having a structure in place for maybe those different levels would be very helpful” (interview F).*

### Future ideas for PPI

#### Method of involvement (description)

All study participants expressed the desire to have more PPI in the education of healthcare professionals. The concept of storytelling, where people talk about their experiences was popular along with co-teaching. There were mixed views in relation to PPI in curricula design, however, the majority reported that with guidance PPI could be of great value.

*“They should be involved in the planning stages, they should be on advisory boards, and so forth to feedback their experiences and their desires for how education should be happening. They should be involved in curriculum design, they should be involved in setting up programmes and actually helping to teach students…as bona fide teachers who are involved in setting up the programme, assessing work and marking and examining students as well.” (interview C).*

There was a deep desire by focus group participants to move away from high stress, time-pressurized situations, such as OSCEs, and have PPI sessions in a more relaxed environment, where they could have a personal, less formal, conversation with students. This was not expressed in interviews. Study participants from both cohorts voiced the importance of being on equal terms with the students. Regular feedback from all participants to help improve PPI was considered important by everyone.

 The common theme that focus group participants believed could be improved on through PPI was communication, knowing how to communicate with people with disabilities and empathy, so that students can get some understanding of what day to day life is like when living with or caring for those with chronic illnesses. They wanted to break down the barriers between healthcare professionals and patients and enhance equality. They believed that caring for patients is about involving the patient in their own care and:

*“Seeing the whole person rather than just dealing with a condition that’s in front of them” (focus group participant R).*

“We *want to be at the table, not on the menu” (focus group participant S).*

#### Timing and duration

Staff believed that PPI needs to be embedded throughout the curriculum.

*“I don’t see why PPI shouldn’t be there from the very get go” (interview E).*

Focus group participants mainly focused on the practicality of regular PPI and the duration and timing. Many focus group participants emphasized the challenges of getting to the university and suggested either half day or full day events to make their journey to the site meaningful.

#### Recruitment methodology

Recruitment was also discussed. Staff were satisfied to recruit patients and members of the public though hospitals as inpatients and in general practice, with some suggesting that we could have more of a partnership with patient advocate associations and use these groups as a method of recruitment. Many focus group participants were satisfied to be recruited for PPI events in a similar manner to how they were recruited for this study, i.e., through patient advocate groups or through their practitioner. Some suggested that there could be an application to become a patient educator on the university website where they could pick and volunteer for certain events. Others mentioned that multiple channels are needed to build a patient database with varied socioeconomic demographics. Popular radio shows were also suggested as a means of raising awareness of PPI and to use it to recruit participants.

*“If you have conditions that’s fairly rare and you’re attending your GP and they’re aware of this program here, they may say, would you be prepared to get involved in PPI because what you have is might be a value in the education system” (focus group participant Q).*

*“Another useful group of organizations that might help academic institutions identify people who might be suitable and add value would be the patient advocacy groups themselves” (interview F).*

## Discussion

This study examined key stakeholders’ views, experiences and expectations of patient and public involvement (PPI) including the nature of the involvement and requirements for partnership. The five themes that emerged from these focus groups and interviews provide an insight into what is currently happening in PPI in education, what could be going wrong, what is going well and how these events could be improved. This study provides novel insights by comparing and contrasting the views of two group of key stakeholders in PPI.

### Experiences of patients, members of the public and members of faculty with PPI

Members of the public are currently involved in education, but their level of involvement is generally low and there is a lack of meaningful engagement in healthcare education. Towle, et al. reported similar findings in their study, where they reported a lack of sustainable involvement in education that possess authentic partnerships with patients at an institutional level [3]. Study participants were mainly involved in OSCE exams where their feedback was not sought. Some patient and public participants were more involved in education and participated as storytellers or in workshops with students, and no participants had been involved in curricula design, although there are opportunities for this at the university. The positive experiences of PPI participants were encouraging, with study participants saying how those involved in such events found it social, enjoyable and invaluable to student learning. The negative experiences expressed were also useful as a guide to how events could be improved in the future. Lack of involvement was a major concern, with some questioning whether their input had any value, others also found that the high stress atmosphere of OSCEs made it less enjoyable and made them feel nervous or uncomfortable at times. The desire for patient and members of the public become more involved in education, is echoed in the literature with many authors describing the value it adds to education [7, 14].

### Views and expectation of patients, members of the public and members of faculty of PPI

Wykurz and Kelly concluded from their systematic review, that when patients are trained, supported and paid they could become colleagues in the education of healthcare professionals rather than an educational resource [28–30]. The need for support and payment was deemed important by all study participants. Most preferred the concept of informal training rather than formal training. This reflected perceived needs, and a concern that formal training would make people less representative. Harrison, et al. similarly suggested that training could prevent PPI participants from being the average representative from that population [18]. This is in line with “professionalization paradox”, which asserts that if a participant receives training, they will achieve a level of professional socialisation that may compromise their status as the average representative [31–33]. Although most participants believed that there was no need for formalised training; good guidance and support was considered essential. Molley, et al. similarly concluded that early discussion of patient expectation and clear communication about the projects direction is required to promote continued success in partnership between health professionals and patients [34]. Training might, however, be required to achieve higher levels of involvement [6]. Despite the reported importance of feedback in PPI, both on participants performance and event organisation, very few studies have attempted to answer ‘how’ or ‘why’ a particular intervention worked [6]. Addressing participant expectations of the outcomes and support before the beginning of the process could help manage expectations. This, along with regular feedback, and acknowledging involvement, are essential to sustainability [4, 19]. Most participants believed that creating a community of PPI participants was important.

The barriers to PPI highlighted in this study show the potential challenges to be overcome. In relation to patient and public participants themselves, the main barriers were availability and illness, which could be mitigated through flexibility in scheduling PPI and increasing the pool of people willing to become involved. This would also help to increase diversity. Confidence and fear of public speaking was also a recognised barrier to becoming more involved that could be addressed through appropriate guidance, support, and matching events to participant preferences. Staff also recognised that limited understanding of PPI was a barrier to sustainable PPI, which could be addressed through appropriate training for academic staff. Incorporating PPI into teaching and learning strategies was suggested as institutional driver for increased inclusion. General lack of awareness of PPI among academics is a well reported barrier in the literature [3, 7].

Cost and sustainability of PPI programmes are two aspects of evaluation of PPI programmes that have not been addressed to any great extent in the literature [6, 35]. A better understanding of costs and benefits would increase incentives for people to be involved and reduce barriers to participation [36].Variations in the payment received by patient and public participants is widely reported, with Towle, et al. reporting a variation of payment models from no payment, expenses only or an hourly rate [3, 37]. Pizzo, et al. proposed that a better economic evaluation will result in better targeting of resources and more effective planning, thus increasing the likelihood of a more meaningful PPI, while avoiding tokenism [37]. Payment was considered important for all participants, not as a primary motivation factor but rather to show that their participation is valued and to include those who may not be able to afford to participate in events. Gordon, et al.’s reported that payment may increase the numbers of PPI participants, thus increasing diversity of conditions and backgrounds. However, they also questioned the impact payment may have on authentic involvement and challenges for patients who are receiving governmental state benefits [6]. INVOLVE (UK National Advisory Group promoting PPI) and the Oxford Academic Health Science Network provides a guide for payment of PPI participants in research which could be a useful resource when deciding how much is suitable for participants based on their level of involvement and a step towards standardization of payments [38, 39]. Along with payment to cover expenses, hospitality in the form of providing refreshments was considered very important as it makes participants feel valued and makes the event more social and enjoyable.

Primary incentives for PPI participants included feeling that they are ‘giving back’ for care they received in their respective healthcare settings, they are helping to educate the future healthcare professionals and improve healthcare services. People also enjoyed activities that were sociable. Some studies have suggested recognition of PPI participants through the application of a formal academic title [3]. This was not considered a major incentive by participants although some appreciated being considered an educator or ‘expert by experience’.

Gordon, et al. reported in their systematic review how patient-led teaching opportunities can cover a wide diversity of topics including storytelling, history taking, curricula design and inter-professional education [6]. The preference was for more opportunities for informal PPI with small groups of students. The concept of patients acting as storytellers and co-teachers where patients interact with students are involved in assessments was advocated by all. Most believed that PPI could add value to curricular design and many patient and public participants indicated they would be happy to be involved if adequate support was provided.

### Limitations

Patients and public were not involved in the conceptualisation of the study, which is the optimal for PPI in research, but were involved in data interpretation and writing the paper. The staff interviewed had some involvement with PPI, either through research or education, and therefore there was a potential for bias, although not obvious in the data. Nevertheless, despite the limitations this study provides educational institutions with valuable insights into the perception of PPI in healthcare education.

### Implication for future practice/ research

The findings from this study provide insight into the elements that are needed in the development of a strategic framework, which is essential to obtain sustainable events throughout educational institutions and move away from the current strategy of isolated PPI events with low levels of involvement. This must be formed at an institutional level so that PPI becomes a core principle of health professional education. There needs to be emphasis on building relationships to sustain authentic PPI.

## Conclusions

 All stakeholders agreed that there is a need for more strategic and formal involvement of patients and the public to create a culture of best practice in PPI. This requires institutional vision and investment. There needs to be a strategic framework to ensure that PPI is embedded throughout healthcare education programmes at an institutional level and working towards national level. Appropriate guidance and support must be provided, with training if required for higher levels of involvement. Ultimately, the key to authentic and sustainable PPI is building relationships.

## Supplementary Information

**Additional file 1.**

**Additional file 2.**

## Data Availability

The data that support the findings of this study are available on request from the corresponding author (MC). The data are not publicly available due to them containing information that could compromise research participant privacy.

## Abbreviations

BEME: Best Evidence Medical Education
COREQ: Consolidated criteria for Reporting Qualitative research
OSCE: Objective Structured Clinical Examinations
PPI: Patient and public Involvement
RCSI: Royal College of Surgeons in Ireland
SOP: standard operating procedures
