# Supplementary material for: Key stakeholders’ views, experiences and expectations of patient and public involvement in healthcare professions’ education: a qualitative study
Source: BMC Med Educ. 2022 Apr 22;22:305. doi: 10.1186/s12909-022-03373-z (PMC9026974; doi:10.1186/s12909-022-03373-z)
Supplement: Supplementary file 2 — Additional file 2. [file 12909_2022_3373_MOESM2_ESM.docx]

Additional file 2: Theme sheet for focus group and interview discussions.

| **Theme sheet for Focus groups** | - Has anyone been involved in educating healthcare students, and if so could you share your experience? - What has, or would, motivate you to become involved in educating our students? - How could educational institutions best recognize the contribution that patients and the public make to educating students? - Do you think training for PPI participants is necessary, and if so how can this training be best delivered? - What would make it easier to be involved in educating students? - What might prevent you from becoming more involved in education? - How can we get patients and members of the public more involved in educating healthcare students? |
| --- | --- |
| **Theme sheet for interviews** | - How do you think patients and the public can best be involved in the education of healthcare professionals? - Do you think training for PPI participants is necessary, and if so how can this training be best delivered? - What do you think would motivate patient and public participants to become involved in PPI? - What are the facilitators and barriers for academics becoming involved in PPI |
